# A deep learning framework for identifying essential proteins based on multiple biological information

**DOI:** 10.1186/s12859-022-04868-8

**Published:** 2022-08-04

**Authors:** Yi Yue, Chen Ye, Pei-Yun Peng, Hui-Xin Zhai, Iftikhar Ahmad, Chuan Xia, Yun-Zhi Wu, You-Hua Zhang

**Affiliations:** 1 Anhui Provincial Engineering Laboratory for Beidou Precision Agriculture Information, Anhui Agricultural University, Hefei, 230036 China; 2 School of Information and Computer, Anhui Agricultural University, Hefei, 230036 China; 3 School of Life Sciences, Anhui Agricultural University, Hefei, 230036 China; 4 State Key Laboratory of Tea Plant Biology and Utilization, Anhui Agricultural University, Hefei, 230036 China

**Keywords:** Essential protein, Deep learning, Protein–protein interaction network, Subcellular localization, Gene expression

## Abstract

**Background:**

Essential Proteins are demonstrated to exert vital functions on cellular processes and are indispensable for the survival and reproduction of the organism. Traditional centrality methods perform poorly on complex protein–protein interaction (PPI) networks. Machine learning approaches based on high-throughput data lack the exploitation of the temporal and spatial dimensions of biological information.

**Results:**

We put forward a deep learning framework to predict essential proteins by integrating features obtained from the PPI network, subcellular localization, and gene expression profiles. In our model, the node2vec method is applied to learn continuous feature representations for proteins in the PPI network, which capture the diversity of connectivity patterns in the network. The concept of depthwise separable convolution is employed on gene expression profiles to extract properties and observe the trends of gene expression over time under different experimental conditions. Subcellular localization information is mapped into a long one-dimensional vector to capture its characteristics. Additionally, we use a sampling method to mitigate the impact of imbalanced learning when training the model. With experiments carried out on the data of Saccharomyces cerevisiae, results show that our model outperforms traditional centrality methods and machine learning methods. Likewise, the comparative experiments have manifested that our process of various biological information is preferable.

**Conclusions:**

Our proposed deep learning framework effectively identifies essential proteins by integrating multiple biological data, proving a broader selection of subcellular localization information significantly improves the results of prediction and depthwise separable convolution implemented on gene expression profiles enhances the performance.

## Introduction

Essential proteins are closely related to the metabolism, differentiation, and apoptosis of cells, which are indispensable for the survival and reproduction of organisms [1]. They help understand the minimum requirements for cell life and the mechanisms of cell growth regulation, which are vital to the discovery of human disease-causing genes and defense against human pathogens [2]. Furthermore, they are instrumental in drug synthesis to find potential targets for new antibiotics in pathogenic organisms [3]. In the early stage, methods for identifying essential proteins include single-gene knockout [4], RNA interference [5], conditional knockout [6], etc., which are usually resource-intensive and time-consuming. With the accumulation of high-throughput data, and the completion of extensive proteome sequencing projects, technologies such as yeast two-hybrid systems [7], affinity purification [8], and microarray gene expression profiling [9] have yielded a wealth of protein interaction information. Consequently, computational methods for identifying essential proteins develop rapidly, reducing experimental costs and improving identification efficiency [10].

Studies have shown that the importance of a protein is closely related to its topological characteristic in the protein–protein interaction (PPI) network. Jeong et al. [1] are the first to propose a topology-based method, degree centrality, judging the necessity of a node based on the number of its adjacency node. Afterward, various centrality methods based on other topological properties arise, while identifying essential proteins merely using the PPI network is insufficient. Researchers found that essential proteins tend to form highly connected clusters, hence, they began to focus on the relationship between the importance of proteins and their aggregation [11], which makes gene expression profiles one of the biological information that researchers value. PeC, proposed by Li et al. [12], determines a protein’s essentiality based on its connectivity and whether it has a high probability of being co-clustered and co-expressed with its neighbors. Another method, WDC [13], units the Pearson correlation coefficient to measure the gene expression profiles and the edge clustering coefficient to weigh the PPI network to identify the essential proteins. Lately, JDC [14] refers to the fluctuations problem in gene expression data and offers a dynamic threshold method to binarize it, then combines the degree centrality and Jaccard similarity index to calculate the JDC score for each protein in the PPI network. Furthermore, studies on the construction of the PPI network have emerged. TGSO [15] firstly adopts the node density measurement method for complex networks to build a protein aggregation degree network, then simultaneously utilizes biological information to construct a protein co-expression interactive network and a protein co-localization interaction network. The three networks are integrated as a comprehensive PPI network and the score of the protein is calculated iteratively based on the homology information. Another prediction model based on the construction of the weighted PPI network, WPDINM [16], is presented by Meng et al. Different from TGSO, this method first combines gene expression data and topological information to construct a weighted PPI network, and then simultaneously builds a domain-domain interaction network. Finally, they integrate two networks as a protein-domain interaction network. The criticality of proteins is estimated with a novel PageRank-based iterative algorithm using the subcellular localization and orthologous information.

Recently, many machine learning and deep learning methods have been applied to predict essential proteins. Researchers generally integrate the PPI network with other biological information, including gene expression profiles, subcellular localization, Gene ontology, etc. With diverse topological and biological characteristics, selecting suitable features to predict essential proteins is one concern. Utilizing 26 features composed of centrality indexes and subcellular localization information as original feature space, Zhong et al. [17] put forward a feature selection method based on SVM-RFE to predict essential proteins. Another feature engineering method proposed by them, XGBGEMF [18], uses the same features mentioned above to construct composite characteristics, obtaining a better subset to predict essential proteins. Meanwhile, node2vec [19] technique, which has been commonly used in deep learning methods to extract topological characteristics from PPI networks, presents a new perspective of automatically feature learning without any manual selection. Zeng et al. construct a deep learning framework DeepEP [20], which treats gene expression profiles as images and uses CNN to extract features. They invoke node2vec to extract topological characteristics from the PPI network and concatenate two biological features together. A sampling method is also used to alleviate the impact of unbalanced learning in their research, which helps effectively in prediction tasks. Considering the sequential characteristics of gene expression profiles, they further raised a method [21], which applies bidirectional long short-term memory cells to capture its non-local relationships. Zhang et al. present a deep learning method, DeepHE [22], to predict essential genes accurately, by integrating 89 sequence features derived from DNA sequence and protein sequence with network embedding features learned from the PPI network to train a multilayer neural network, They also use a cost-sensitive technique to address the imbalanced learning problem.

Concerning the traditional centrality method, in the first place, denoting the topological characteristics using one centrality index scalar is incomplete. Secondly, since the noise in the PPI network is gradually increasing, it is inadequate to identify essential proteins merely depending on the PPI network. Some feature engineering methods may filter out a better subset, while the initial feature space is selected manually, and it is uncertain whether these centrality features can fully represent the topological properties of network nodes. Several machine learning and deep learning methods combine gene expression profiles and subcellular localization data with the PPI network to predict essential proteins. They generally select 11 or 17 types of pivotal subcellular localization to construct feature space, while these common methods fail to adequately cover the proteins in the PPI network. Many gene series matrices provide the data with time courses and control experiments. Some deep learning methods have studied the trend over the time course, while few of them observe the different conditions in the meantime.

To tackle the problems mentioned above, we proposed a deep learning method, combining the PPI network, gene expression profile, and subcellular localization to predict essential proteins. We use the node2vec technique to learn a mapping of nodes to a low-dimensional space of features to obtain topological properties in the PPI network. Different from some existing deep learning treatments which generally target temporal features of gene expression profiles to extract key proteins, in hope of extracting the patterns of gene expression data that change along temporality under different experimental conditions, we chose an experimental series matrix that provides both disparate treatment and temporal data. The concept of depthwise separable convolution is applied to gene expression profiles to understand the pattern under different experimental environments. We use 1-dimensional convolution (Conv1D) to learn temporal characteristics of gene expression separately, and then use pointwise convolution to merge the features from two environments. In addition, unlike the common disposal of subcellular localization in machine learning and deep learning methods, we did not select several common subcellular localizations. To ensure most samples are characterized by subcellular localization, we rank the number of proteins corresponding to each subcellular localization in the PPI network and then selected the top 1024 of them as feature space. From the integrated channel, we select a broader scope of subcellular localization and map it as a long vector, covering 96.34% of proteins in the PPI network. Furthermore, a sampling method is also employed to address the unbalanced learning problem due to the essential proteins accounting for only a minor proportion. Plenty of experiments are carried out on the dataset of Saccharomyces cerevisiae to validate the performance of our model. To begin with, we compare the disposal of every biological information partly to confirm whether our processing is superior. Then, we split and recombine the biological information to observe the contribution of each element to the classification. Finally, we measure the performance between the balanced dataset and raw dataset and compare our method with ten centrality methods, six machine learning methods, and one deep learning method, which turns out our model outperforms these measures.

## Methods

### Overview

**Fig. 1:**
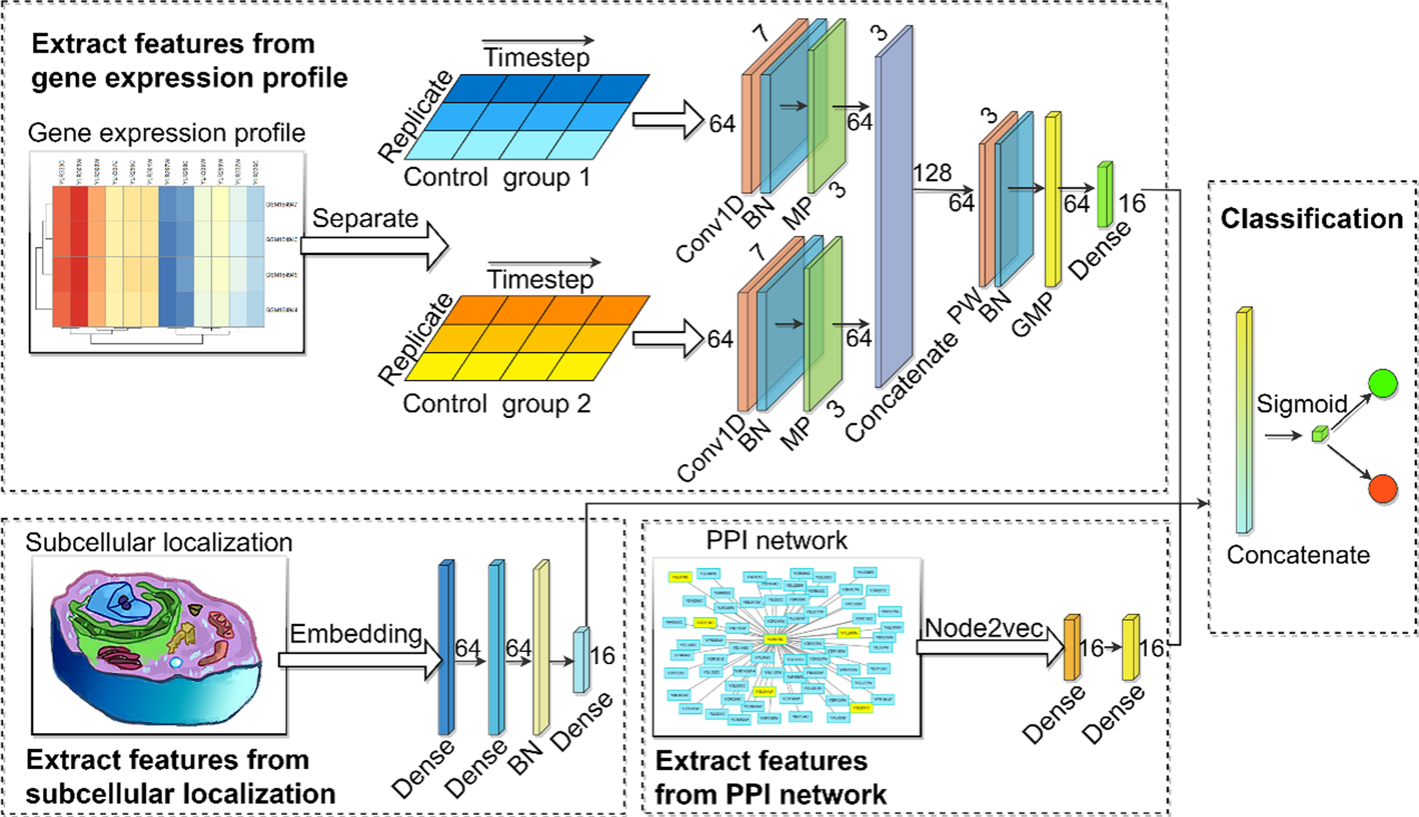
General structure of our framework. *Conv1D* 1-dimensional convolution, *BN* batch normalization, *MP* max pooling, *PW* pointwise convolution, *GMP* global max pooling

Figure 1 displays the overall structure of the proposed deep learning framework, mainly consisting of two parts: the feature extraction component and the classification component. Our model includes three inputs, namely gene expression profile, subcellular localization, and PPI network. In the feature extraction section, we separate the gene expression profiles according to different experimental environments and then implement Conv1D on the data respectively to learn the features along the time step, followed by a normalization operation and a max pooling process to prevent overfitting. After that, we concatenate the outputs of two channels to transmit to a pointwise convolution to integrate information from the different conditions. A global max pooling operation is utilized to flatten the data, and a fully connected layer is used to condense it. As for the PPI network, the node2vec technique is used to learn the topological properties of every protein, and two fully connected layers are applied to the embedding output. Subcellular localization data is mapped into a long 1-dimensional vector, and three fully-connected layers are conducted to enrich the feature. Finally, we combine the result from three biological information as the input for the classification part. Given the minor proportion of essential proteins, a sampling method is utilized to address the unbalanced learning problem.

### Features derived from gene expression profiles

Observing the gene expression profile, we found the data along the time course between replicate samples appears to have a higher relevance. Still, the data along the time course between different conditions seem more independent. GSE7645 [23] is selected as our object of analysis, concluding with no-treated cultures as control groups and CHP exposed cultures as observation groups with three replicate samples set each. The experiment studied the genome-wide temporal response of the yeast S. cerevisiae to oxidative stress, which is a well-known biological process that occurs in all respiring cells and is involved in pathophysiological processes such as aging and apoptosis, while essential proteins are closely related to the cell metabolism, differentiation, and apoptosis [10]. In addition, the experiment provides expression data over time for different environments, satisfying the conditions of our model.

1 $$x = \frac{{\sum \left( {x - m_{x} } \right)\left( {y - m_{y} } \right)}}{{\sqrt {\sum \left( {x - m_{x} } \right)^{2} \sum \left( {y - m_{y} } \right)^{2} } }}$$

**Fig. 2:**
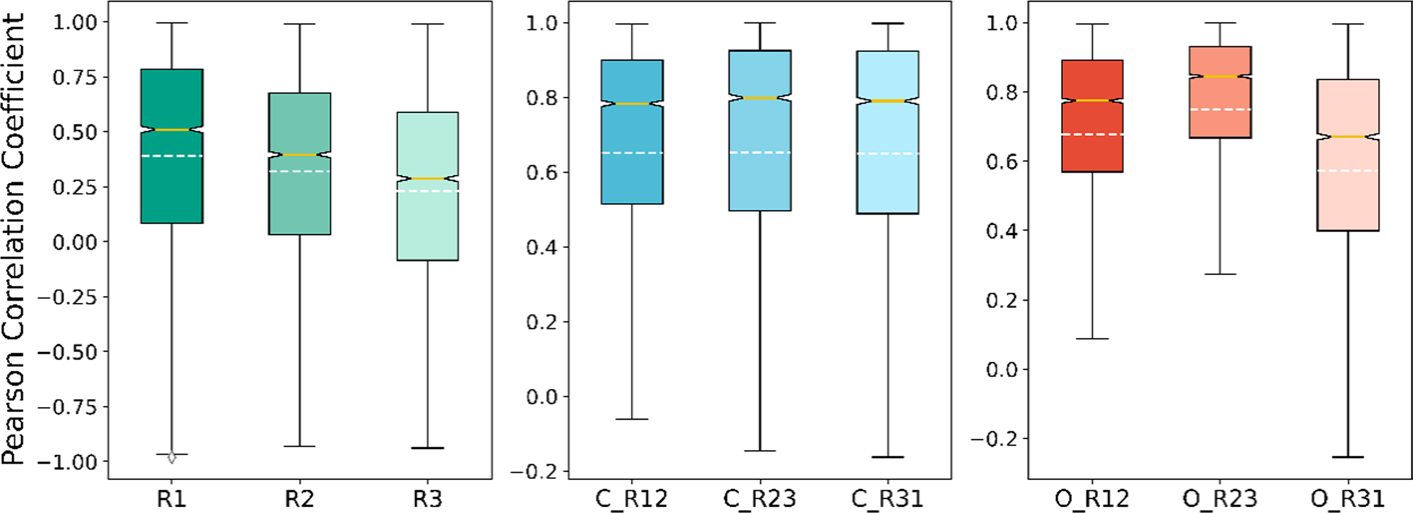
The PCC between replicate samples and different conditions in the gene expression profiles. *C* control group, *O* observation group, *R1* replicate sample 1, *R2* replicate sample 2, *R3* replicate sample 3

The Pearson correlation coefficient (PCC) [24], which defines as Formula (1), is utilized to measure the relevance between two vectors, x, and y, where m_x_ and m_y_ denotes the mean value of them partly. PCC varies between − 1 and + 1, with 0 implying no correlation, while − 1 or + 1 indicates an exact linear relationship. For every protein, to begin with, we calculate the PCC of the replicate samples between two different environments, then we compute the PCC between three replicate samples in the same environment. As plotted in Fig. 2, we can see the distribution relationship of proteins under different comparisons. Although the values of samples in different environments are relatively discrete, the overall trend is between 0.1 and 0.6. Meanwhile, in the control group and the observation group, the correlation between different replicate samples is steep, in the general range of 0.6–0.9. Due to the replicate sample being an exact copy of a sample, it is reasonable to have a high positive correlation.

To observe changing trends from the aspects of time course and experiment environment, we use the concept of depthwise separable convolution [25]. It applies a single convolutional filter per each input channel, and pointwise convolution is used to create a linear combination of the output of the depthwise convolution. To begin with, assuming the control group and the observation group as different channels, we separate the original data and apply Conv1D to learn the feature of the time course in each channel. The output is displayed in Formula (2), in which C denotes the number of channels—while under current circumstances indicates the number of replicate samples in the individual environment. b and W separately represent bias, and weight.

2 $$Out\left( {C_{{out_{j} }} } \right) = b\left( {C_{{out_{j} }} } \right) + \mathop \sum \limits_{ k = 0}^{{C_{in} - 1}} W\left( {C_{{out_{j} }} ,k} \right) \odot Input\left( k \right)$$

Secondly, batch normalization [26] processing follows to help the model generalize better to new data. The transformation operation is shown in Formula (3). When B denotes the numerical set passed in, μ_B_ and σ_B_^2^ represent the mean value and variance of B, which are applied to maintain the mean output close to 0 and the standard deviation output close to 1. With γ and β referring to the trainable parameters, y_i_ implies the output for every number in B.

3 $$y_{i} = \gamma \frac{{x_{i} - \mu_{B} }}{{\sqrt {\sigma_{B}^{2} + \varepsilon } }} + \beta$$

Notably, batch normalization works differently during training and inference. During training, the mean and variance are respectively the mean and variance of the corresponding dimensions of the data in the batch. During prediction, the mean and variance are calculated based on the expectations of all batches.

At the end of the separate disposal, we use the max pooling operation to process the results above, with the pool size set as 2. Subsequently, the outputs of two channels are concatenated together to go through a pointwise convolution [27], which computes features that mix information from the channels of the input tensor and not mix information across time since it is looking at one tile at a time, where they contribute to factoring out channel-wise feature learning and time-wise feature learning. Ultimately, we use the global max pooling process to reduce data dimension, and a dense layer with hidden units as 16 is employed to produce the output. To demonstrate that the model can learn different patterns under different controls, we selected a protein and visualized its gene expression profile data processing.

**Fig. 3:**
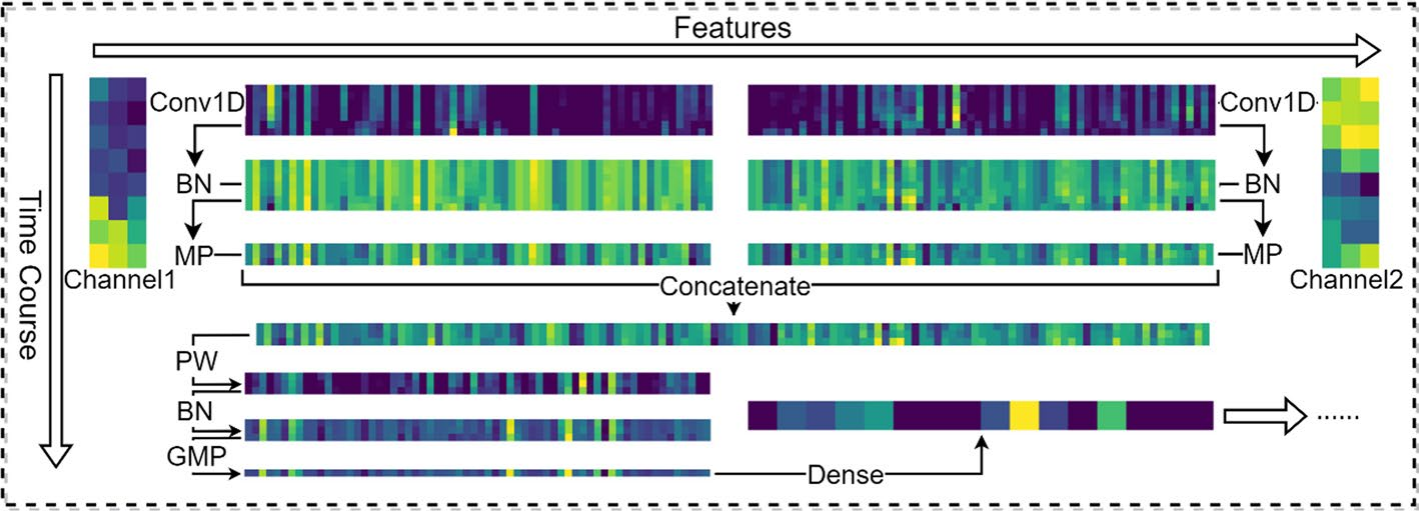
Visualization of gene expression profile processing. *Conv1D* 1-dimensional convolution, *BN* batch normalization, *MP* max pooling, *PW* pointwise convolution, *GMP* global max pooling

Figure 3 displays the visualization of convolution processing under two channels. It can be seen that the gene expression data in the two environments are distinct, while the data between replicate samples are similar. After independent processing of the two channels, the output results of the middle layer prove that the patterns extracted are discrepant, which is beneficial to model learning. Since the more multifaceted data patterns can be observed, the more the model can learn.

### Features extracted from subcellular localization

Some machine learning and deep learning methods employ commonly used subcellular localizations to build feature space [17, 18, 21]. Applying the method in the paper [21], it turns out that 19.17% of proteins in our PPI network possess none of these features. Therefore, hoping that the feature could cover most proteins in our PPI network, we download the integrated channel data from the COMPARTMENTS database [28], which integrates data from different channels to obtain a final confidence index. The confidence index is provided by the COMPARTMENTS database for every subcellular location-protein entry, indicating the reliability of the types and sources of localization information. The higher the confidence score, the more reliable the confidence of the subcellular localization-protein entity evidence. Next, we counted the number of proteins corresponding to all subcellular localization in the file and then sorted the subcellular localization in descending order according to the number. Finally, select the top 1024 subcellular localizations in the ranking result as the feature space, containing 96.34% proteins in the network. At the same time, we maintain the representativeness of every subcellular localization.

**Fig. 4:**
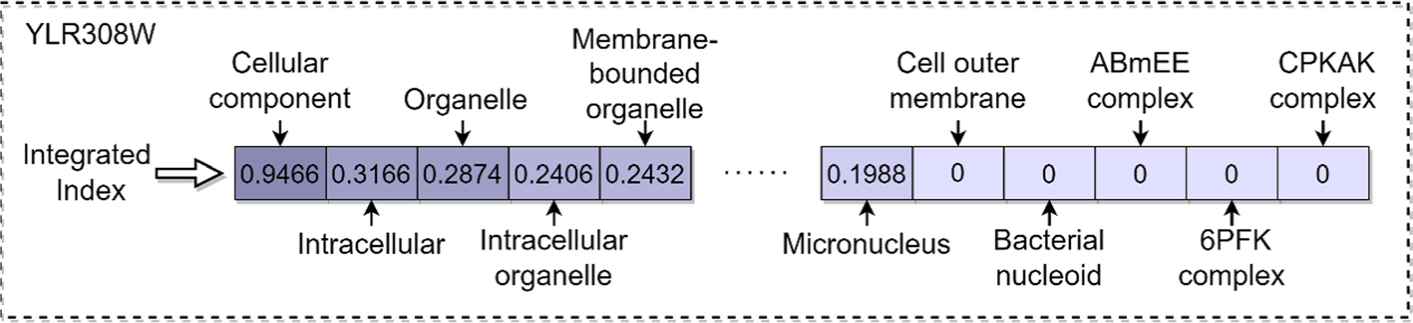
The process of subcellular localization data for protein YLR308W. *ABmEE complex* Apolipoprotein B mRNA editing enzyme complex, *CPKAK complex* cyclin-dependent protein kinase activating kinase holoenzyme complex, *6PFK complex* 6-phospho fructose kinase complex

As shown in Fig. 4, the subcellular localization information of each protein is mapped into a long one-dimensional vector, and the confidence score provided by the integrated channel is normalized. We use three fully connected layers to process it. The first two layers set 64 hidden units to compress the output length from 1024 to 64, then batch normalization is applied to prevent overfitting, and the last layer sets 16 hidden units as the output of the feature. We set the outcome of each component as a vector with a length of 16 so as not to interfere with the weight of each part since the contribution of each component is not explicit yet.

4 $$f\left( x \right) = max\left( {0,x} \right)$$

The rectifier linear unit function (4), which can access a much richer hypothesis space that would benefit from deep representations, is employed as the activation function. Formula (5, 6) displays the calculation for each layer. With I, W, and b respectively denoting the input, weight matrix, and bias of this layer, the adjusted signal is obtained with initial weight, then the activation function is utilized to judge each element in the matrix to acquire the output y.

5 $$Z = W \cdot I + b$$

6 $$y = f\left( Z \right)$$

### Features learning from PPI network

In traditional centrality methods, the score of one protein in the PPI network is generally calculated based on certain scoring criteria. These calculation standards indicate unique theory, so their representativeness is relatively limited. With the increase in network scale, it is insufficient to measure the topological properties with one scalar score. Meanwhile, the feature engineering methods also require manually selecting the original feature space. After the optimization, the best subset usually contains several centrality indexes. Hence, we require a method that not only learns the topological characteristics automatically but also gets richer representations.

Node2vec [19], a semi-supervised algorithm for scalable feature learning in networks, automates the whole process by casting feature extraction as a representation learning problem that requires no hand-engineered features. Researchers formulate feature learning in the network as a maximum likelihood optimization problem, and stochastic gradient descent is applied to optimize a custom graph-based objective function. Skip-Gram architecture, which is widely used in the field of neuro-linguistic programming, is extended on the network to derive feature representations. Different from the linear nature of the text, a richer notion of the neighborhood for nodes in the network is required. Thus, researchers propose a flexible biased random walk procedure that samples many different neighborhoods of a given source node. For a given network G = (V, E), where V and E respectively denote the set of vertexes and edges, a random walk of fixed length l for source node u ∈ V is simulated. During the process, the probability of the i_th_ node in the walk observes the distribution below:

7 $$P\left\{ {c_{i} = x{|}c_{i - 1} = v} \right\} = \left\{ {\begin{array}{*{20}l} {\frac{{\pi_{vx} }}{Z}} \hfill & {if\; \left( {v,x} \right) < 0} \hfill \\ x \hfill & {otherwise} \hfill \\ \end{array} } \right.$$

where π_vx_ is the unnormalized transition probability between nodes v and x, and Z is the normalizing constant. The second-order random walk with parameters p and q is utilized to guide the walk. When the walk just transitioned from node t to node v, the evaluation of the next step depends on the probability of π_vx_ = α_pq_(t, x)·w_vx_, where

8 $$\alpha_{pq} \left( {t,x} \right) = \left\{ {\begin{array}{*{20}l} \frac{1}{p} \hfill & {if\; d_{tx} = 0} \hfill \\ 1 \hfill & {if \;d_{tx} = 1} \hfill \\ \frac{1}{q} \hfill & {if\; d_{tx} = 2} \hfill \\ \end{array} } \right.$$

and d_tx_ denotes the shortest path distance between node t and x. In our experiment, the dimension of feature representations is set as 64. For the random walk process, we set the length as 20 and the number of walks as 10. In the meanwhile, the context size of the optimizer is also set to 10, and the other parameters remain the defaults. After that, two fully connected layers are called on the feature generated by the node2vec technique from the PPI network, with hidden units set as 64 and 16, respectively.

### Data and materials

The proposed model consists of three inputs: protein–protein interaction networks, gene expression profiles, and subcellular localization information. We also require essential proteins to label the dataset.

PPI networks are derived from BioGRID [29] database, a regularly updated public database that archives and disseminates genetic and protein interaction data from model organisms and humans. We downloaded the new version (release 4.4.200, July 2021) of Saccharomyces cerevisiae data as the PPI networks. In our experiment, physical interaction edges are adopted only. Besides, self-loops and duplicate edges are removed to reduce data noise. The PPI network has 127,581 edges and 5988 nodes after processing, with an average degree of 21.31.

Essential proteins are retrieved from different sources, with DEG [30] database offering 1109 essential proteins and OGEE [31] database providing 946 ones. Information from two databases is combined, and duplicate records are removed, leaving 1132 proteins in the union set, which takes up 18.90% of the total samples.

Subcellular localization data is extracted from the integrated channel of the COMPARTMENTS [28] database (October 2021), a weekly updated web resource that integrates evidence on protein subcellular localization from manually curated literature, high throughput screens, automatic text mining, and sequence-based prediction methods.

Gene expression profiles are collected from Gene Expression Omnibus, a public functional genomics data repository. We download the series matrix file of GSE7645 [23], a study of expression data for Saccharomyces cerevisiae oxidative stress response. The overall design of the experiment comprises three replicate cultures in the mid-exponential phase exposed to 0.19 mM CHP with three non-treated cultures used as controls, and samples were collected at the time point of 0, 3, 6, 12, 20, 40, 70, 120 min after adding the oxidant respectively. The raw data held 9335 probe records, leaving 5658 entries after matching probe ids from the annotation package. Given that a protein may correspond to more than one probe, we calculated the average maximum expression data as the only result of multiple corresponding entries, leaving 5476 entries after processing, covering 91.45% of the total sample.

Our experiments are performed on TensorFlow [32] version 2.4.0. The filters for all convolution operations are set to 64, and the activation function for all convolution operations is selected as the rectified linear unit function. During training, we set the epoch to 20 and batch size to 64. RMSprop is considered as the optimizer with the learning rate set as 0.001. For the original data set, we first randomly disrupt it. Then 60% of the data is taken as the training set to obtain trainable parameters, 20% as the validation set for hyperparameter tuning, and 20% as the test set, which participates in no training steps, to inspect the ability of the model to predict new samples.

### Evaluation metrics

We evaluate the performance of our method with other methods by comparing the AUC score and AP score, which separately indicate the area under the ROC curve and the area under the PR curve. ROC curve is a graphical plot created by plotting the true positive rate against the false positive rate at various threshold settings. The PR curve refers to a graph with the precision value against the recall value. Additionally, we also employ other metrics to evaluate the model as shown in Formula (9–14), where TP, TN, FP, and FN represent the number of true positives, true negatives, false positives, and false negatives. Accuracy counts the number of correct predictions in all samples. Due to the unbalanced learning problem in our classification task, we further utilize precision and recall. Precision records the number of correct predictions in the samples with positive predictions, denoting the credibility of the model’s judgment on positive prediction, while recall statistics the correct predictions in all positive samples. In our aim of identifying essential proteins, the two metrics are equally important.

9 $$Accuracy = \left( {TP + TN} \right)/\left( {TP + TN + FP + FN} \right)$$

10 $$Precision = TP/\left( {TP + FP} \right)$$

11 $$Recall = TP/\left( {TP + FN} \right)$$

Thus, we apply another metric, the F1 score, which is the harmonic mean of precision and recall. The closer the F1 score to 1, the better the comprehensive performance of the model in terms of recall and precision. At the same time, the value of the f1 score decreases when the value of either the recall or the precision becomes lower.

12 $$F1\; score = 2 \cdot precision \cdot recall/\left( {precision + recall} \right)$$

13 $$Specificity = TF/\left( {TN + FP} \right)$$

14 $$NPV = TN/\left( {TN + FN} \right)$$

Furthermore, we also employ specificity and negative predictive value (NPV). The former represents the proportion of correct predictions in all actual negative samples, and the latter refers to the number of correct predictions in the samples with negative predictions. Notably, the F1 measure, AUC score, and AP score are more important than other metrics in an imbalanced learning problem on the ability to comprehensively provide a classification assessment.

## Result

### Comparison of different processes on gene expression profiles

To demonstrate our separable approach to gene expression profiles is capable of excavating more patterns, we implement several comparative experiments, which reserve different environments sharing the same axis with replicate samples. Considering the time characteristic of gene expression data, we respectively apply Conv1D and RNN-based algorithms, namely long-short term memory (LSTM) [33], gated recurrent unit (GRU) [34], bidirectional LSTM (BiLSTM), and bidirectional GRU (BiGRU) for comparison. Among them, the ordinary Conv1D is used to verify the effectiveness of separation processing. The control group, observation group, and replicate samples are put on the channel axis, with other procedures and configurations remaining the same. Disposals based on RNN are conducted by the TensorFlow instance, in which LSTM and GRU set the unit as 64, and the ones calling the bidirectional operation set the unit as 32. For tests that employ LSTM instance, dropout and recurrent dropout are set to 0.1 and 0.5 partly.

**Fig. 5:**
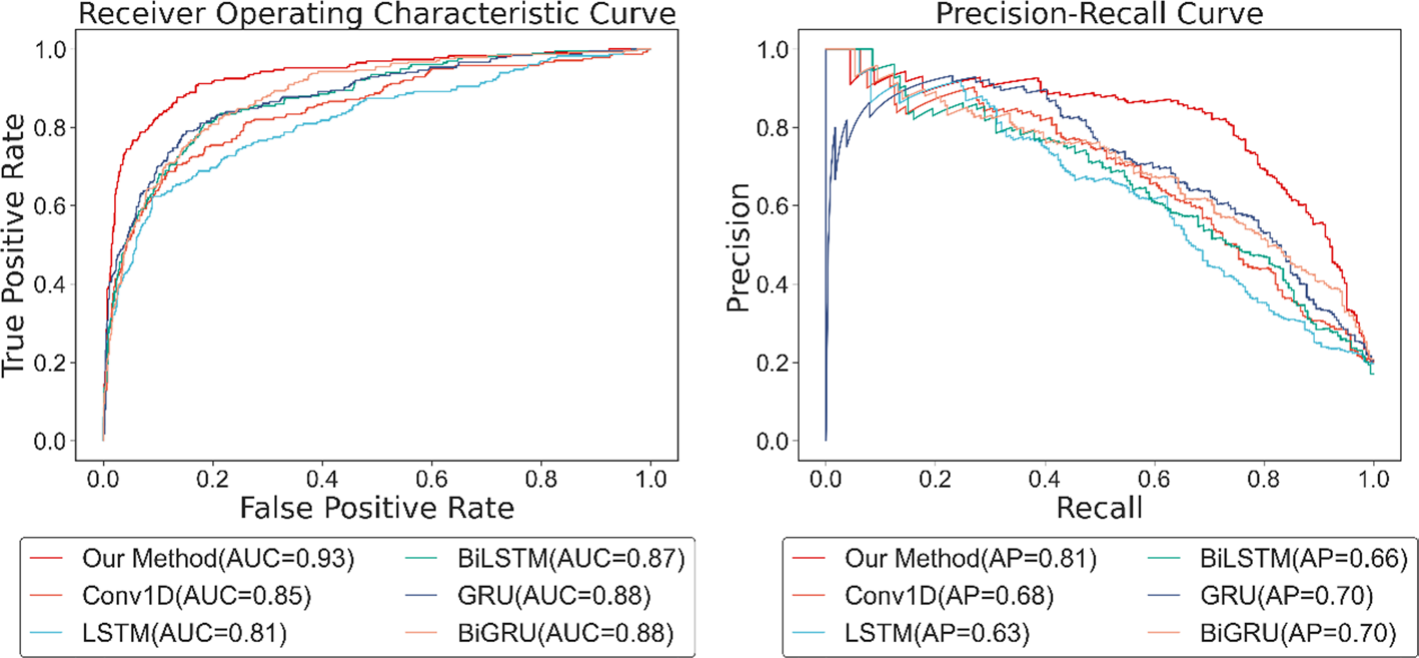
ROC and PR curve of different processes on gene expression profiles

To ensure the reliability of comparison experiments, the six types of treatment keep the output identical in length, and no other parts of the model or training parameters are modified. After implementing the six methods on gene expression profiles, the ROC and PR curves are shown in Fig. 5. With the AUC score of 0.93 and AP score of 0.81, our method performs better, while the ordinary Conv1D has an AUC score of 0.85 and an AP score of 0.68. From the terms of AUC score and AP score, BiLSTM is slightly better than LSTM, while GRU and BiGRU appear the same results. On the whole, the performance of these RNN-based methods is rarely diverse. Apart from this, we also evaluate the six different approaches on other metrics illustrated in Table 1.

**Table 1:** The performance of different processes on gene expression profiles

Method	Accuracy	Precision	Recall	F1 score	Specificity	NPV
Our Method	0.9048	0.7306	0.7885	0.7585	0.9320	0.9496
Conv1D	0.8422	0.5902	0.6833	0.6344	0.8820	0.9175
LSTM	0.8523	0.6311	0.5628	0.5950	0.9214	0.8982
BiLSTM	0.8514	0.5433	0.6900	0.6079	0.8838	0.9343
GRU	0.8606	0.6403	0.6807	0.6599	0.9052	0.9196
BiGRU	0.8581	0.6713	0.5943	0.6304	0.9256	0.8992

Firstly, we find the value of accuracy, precision, recall, f1 score, NPV, and specificity in our method is 6.26%, 14.04%, 10.52%, 12.41%, 5.00%, and 3.21% higher than the ordinary Conv1D method respectively. Secondly, LSTM and BiLSTM have minor differences in performance. Although they are diverse in recall and precision, their F1 score and accuracy are very close. Taking the AUC score and AP score into account, BiLSTM might be slightly better than LSTM, while the bidirectional operation in LSTM has little impact on the results of this experiment. Furthermore, GRU is superior to the methods using LSTM in all aspects, and the utilization of bidirectional hardly improves the performance of GRU. Though the precision and specificity of BiGRU are 3.10% and 2.04% higher than GRU, its other metrics are not as well as GRU. In general, among the RNN-based processing methods, GRU displays the best result. The GRU is like the LSTM with a forget gate, but has fewer parameters than LSTM, as it lacks an output gate, and has been shown to exhibit better performance on certain smaller datasets. Bidirectional operation simply consists of two regular RNNs, each processing input sequence in one direction, then merging their representations, hoping to catch patterns that may have been overlooked by one-direction RNN. Nonetheless, the gene expression profiles used in this experiment own a short timestep of length 8, thus, applying fewer parameters and a simpler mechanism in training is a better choice. The overall result demonstrates that it is recommended to split the channels and process the data separately for gene expression profiles with time courses and multiple conditions.

### Comparison of different selections on subcellular localization

To prove our disposal of subcellular localization is better than other treatments, we adopt the handling in paper [32] as a contrast. To begin with, they downloaded the knowledge channel in the COMPARTMENTS database, then selected cytoskeleton, cytosol, endoplasmic, endosome, extracellular, Golgi, mitochondrion, nucleus, peroxisome, plasma, and vacuole to map the feature for every protein. We implement the same procedures and obtain a vector with a length of 11 from the knowledge channel. Likewise, we also downloaded the integrated channel and select the same 11 subcellular localizations to construct the feature space. In our PPI network, the 11 subcellular localizations from the knowledge channel cover 80.83% of proteins, while those from the integrated channel cover 96.13% of proteins. We want to explore whether the coverage of features has an impact on the performance of the model. Furthermore, to study the relevance between the scope of subcellular localization and the performance of the model, based on the selection method mentioned in the previous section, we select the top 64, 128, 256, 512, and 1024 subcellular localization respectively in the sorting results to construct the feature space, and they all covered 96.34% of the proteins in the PPI network. We remain other parts of the model unchanged to make the comparison results more credible.

**Fig. 6:**
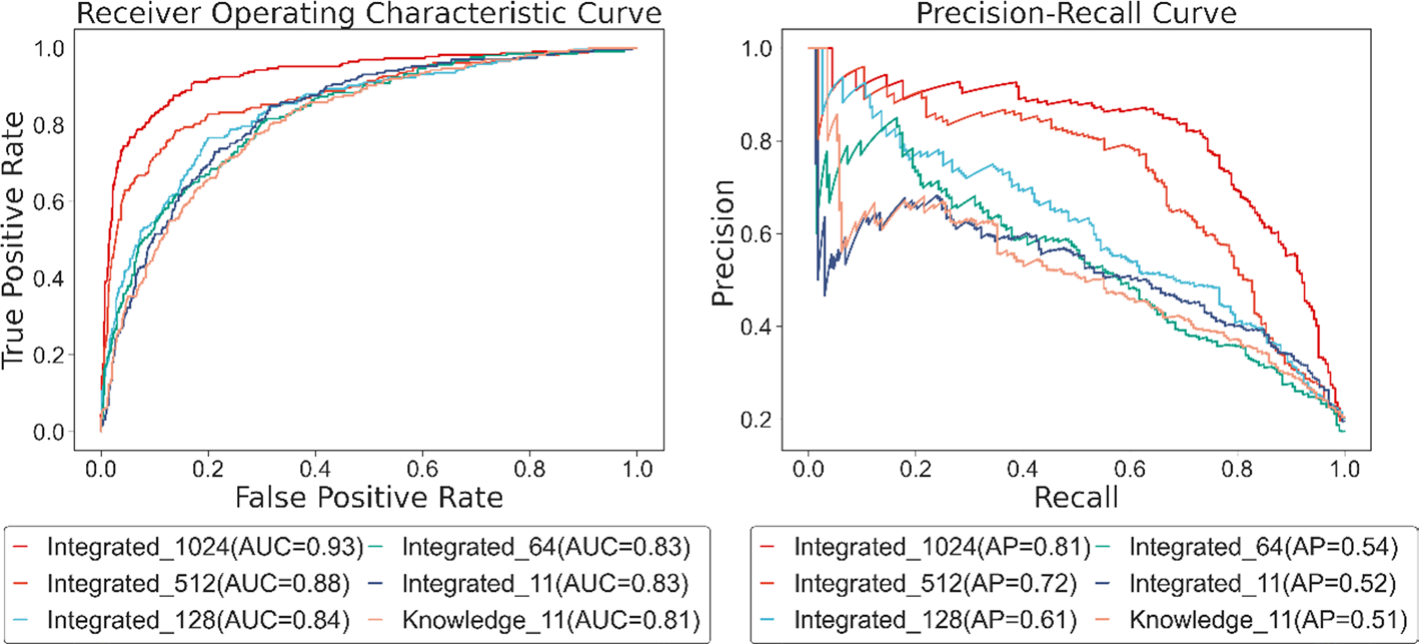
ROC and PR curve of different selections of subcellular localization

Figure 6 reveals the ROC and PR curves of the seven measurements. Employing the same 11 subcellular localizations, the curve of the integrated channel roughly encloses the curve of the knowledge channel, indicating that the coverage of features has an impact on the model. Under the same conditions, the more comprehensive the coverage samples are, the better the performance of the model will be. Applying the knowledge channel, nearly 19.17% of the proteins do not have features, however, the proportion of positive samples in this experiment is only 18.90%, so it is reasonable for the integrated channel to perform better. Although the trend of the curve of Integrated 64 has some twists, from an overall perspective, the length of subcellular localization is approximately in a positive correlation with the performance of the model. There is little difference between Integrated_64 and Integrated_128, thus, we infer that the features between the position of 64 and 128 contribute less to the identification of essential proteins.

As illustrated in Table 2, the performance of seven disposals demonstrates the integrated data retrieved with our method (Integrated_1024), is superior to the feature from the knowledge channel (Knowledge_11), with the value of accuracy, precision, recall, f1 score, specificity, and NPV in our method being 11.52%, 27.21%, 18.16%, 23.58%, 9.99%, and 4.88% exceeds. In the first place, the common method sifts the data from the knowledge channel with 11 subcellular localizations decreasing the supply of other information. More importantly, nearly 20% of proteins possess none of the selected 11 subcellular localization, which causes insufficient coverage that leads to poor performance. In contrast, our method uses combined data extracted from different sources and extends the length of the features considerably, thus providing a richer representation and ensuring that the majority of the sample is included. Additionally, we found that from Integrated 128 to Integrated 1024, the model performance rises steadily, so we judge that there are properties in this section of data that enable the model to observe more aspects. We stop continuing to extend the length of the feature because the subcellular localization at position 1024 in the sorting results corresponds to only 136 proteins in the PPI network. Further adding features that are not representative enough will waste computing power and may not improve the effect of the model.

**Table 2:** The performance of different selections of subcellular localization

Method	Accuracy	Precision	Recall	F1 score	Specificity	NPV
Integrated_1024	0.9048	0.7306	0.7885	0.7585	0.9320	0.9496
Integrated_512	0.8848	0.7304	0.6422	0.6835	0.9431	0.9165
Integrated_256	0.8548	0.6046	0.6943	0.6463	0.8927	0.9251
Integrated_128	0.7988	0.5253	0.7220	0.6081	0.8200	0.9145
Integrated_64	0.8214	0.4966	0.6840	0.5754	0.8509	0.9260
Integrated_11	0.7922	0.4752	0.6567	0.5514	0.8249	0.9087
Knowledge_11	0.7896	0.4585	0.6079	0.5227	0.8321	0.9008

### Comparison of centrality indexes and network embedding features

To validate that the node2vec technique generates a more comprehensive and effective feature representation, ten well-known centrality methods, namely DC, BC, CC, EC, SC, SoECC, ClusterC, MNC, LAC, and LID [1, 11, 35–42], are utilized in the comparison. First, we calculate the ten centrality indices for each protein. Then, we replace the node2vec-generated 1D vector input of the PPI network in our model with centrality values, remaining the disposal process of gene expression profiles and subcellular localization unchanged. Finally, we train the model and evaluate the performance. Moreover, we concatenate the ten indexes together to test whether it provides a preferable result.

**Fig. 7:**
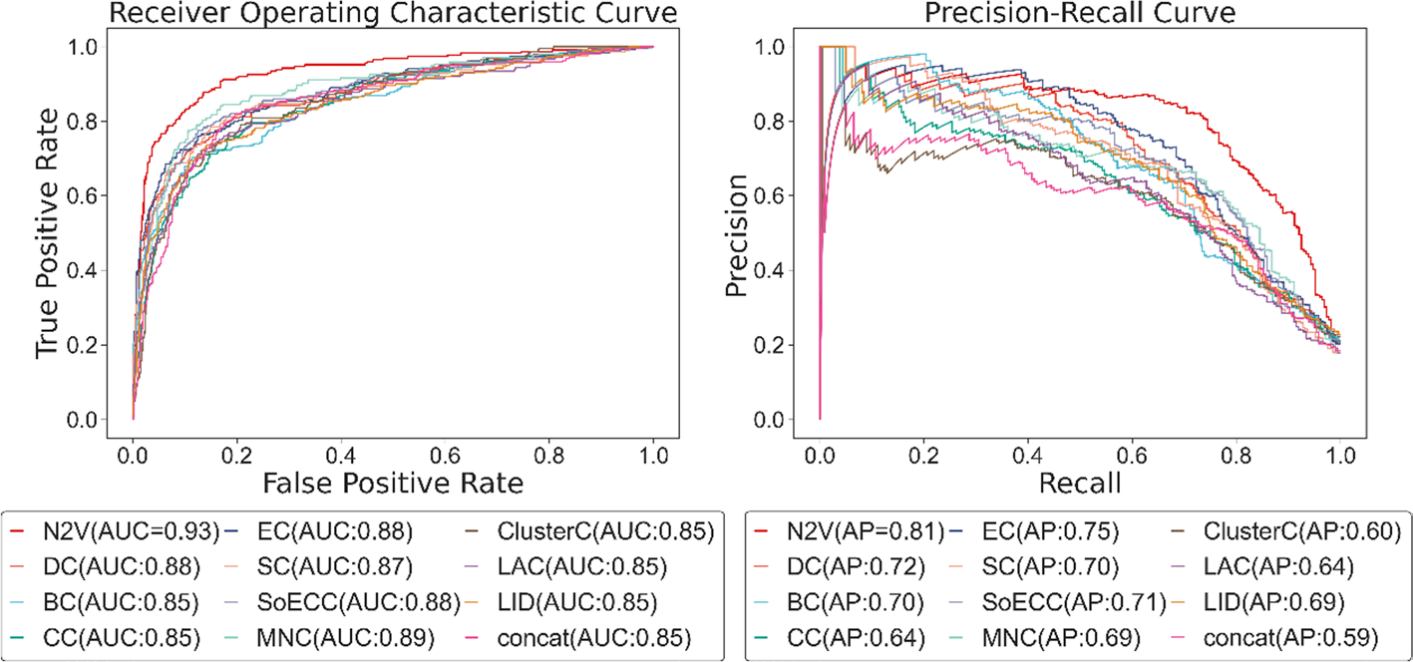
ROC and PR curve of node2vec technique and centrality methods

Except for the input of the PPI network part in the model, we keep the other settings unchanged. In Fig. 7, we plot the ROC and AP curves of the node2vec technique and other methods. We find that the AUC score of the centrality indexes shows little difference from that of node2vec, which is 0.08 less at most, while the AP value has more obvious gaps. Although the PR curve of these methods intersected at begin, the ROC of the node2vec curve is the largest. Intriguingly, using a single centrality index to replace the 64-dimensional vector of node2vec appears little difference, while the feature concatenating ten centrality indexes together reflects mediocre results, which may indicate that simply combining the centrality properties is inadequate to provide a comprehensive feature. We believe that some centrality indexes may be highly correlated, which leads to redundancy problems because the smaller the difference between feature representations, the fewer patterns the model can learn.

Table 3 presents that node2vec performs better than DC, BC, CC, SC, ClusterC, LAC, LID, and the concatenated method in the six metrics. In general, using the index of EC to replace node2vec reflects the best result, with an accuracy of 0.8840 and precision of 0.7011, which is supreme in the comparison of centrality indexes. Even though MNC and SoECC show comparatively high accuracy of 0.8765, 0.8781, and precision of 0.7070, 0.7987, which is 6.81% higher than our method, they possess a low recall of 0.5211 and 0.5064. It is a manifestation of the trade-off between precision and recall, which draws forth the f1 score to consider these two metrics at the same time. With MNC and SoECC owning f1 scores of 0.6000 and 0.6198, we determine that, from the comprehensive level, node2vec and EC outperform MNC and SoECC.

**Table 3:** The performance of node2vec technique and centrality indexes

Method	Accuracy	Precision	Recall	F1 score	Specificity	NPV
Node2vec	0.9048	0.7306	0.7885	0.7585	0.9320	0.9496
DC	0.8656	0.6443	0.6966	0.6694	0.9066	0.9249
BC	0.8589	0.6681	0.6163	0.6412	0.9213	0.9033
CC	0.8406	0.5896	0.6271	0.6078	0.8929	0.9071
EC	0.8840	0.7181	0.6849	0.7011	0.9333	0.9228
SC	0.8581	0.5760	0.6923	0.6288	0.8929	0.9325
MNC	0.8765	0.7070	0.5211	0.6000	0.9533	0.9020
ClusterC	0.8539	0.6079	0.6161	0.6120	0.9086	0.9114
SoECC	0.8781	0.7987	0.5064	0.6198	0.9688	0.8894
LID	0.8573	0.7206	0.5632	0.6323	0.9392	0.8853
LAC	0.8623	0.6054	0.6368	0.6207	0.9108	0.9210
Concatenate	0.8497	0.5714	0.6667	0.6154	0.8900	0.9239

Notably, despite the result of the centrality index being just a scalar, when replacing the representation extracted by node2vec with it, the disparity of outcome is not as significant as we thought. Taking EC, for example, the result of recall is 10.36% less than node2vec, while the other indicators are 5.74% lower at most, which hints that despite centrality methods being proposed long before, treating their indexes as the input of the deep learning model still shows an acceptable result. There is no doubt about the role the PPI network played in classification, yet the small gap in the results between centrality indexes and node2vec arouses the reflection of whether can we go further in the extraction of network features and how much every part contributes to the model capability.

### Comparison of different combinations of features

In the previous three experiments, we separately implement comparison tests on the process of three biological inputs. After demonstrating our treatment is practically more effective, we conduct another experiment to confirm every feature is indispensable and sort out the contribution of each part to the model prediction. We split the three feature extraction components of our model, then test each separately, transmitting the 16-dimensional vector exported by the single part to the classification component to obtain the last result. Moreover, we carry out the recombination among the features, concatenating the output together to pass to the classification section.

**Fig. 8:**
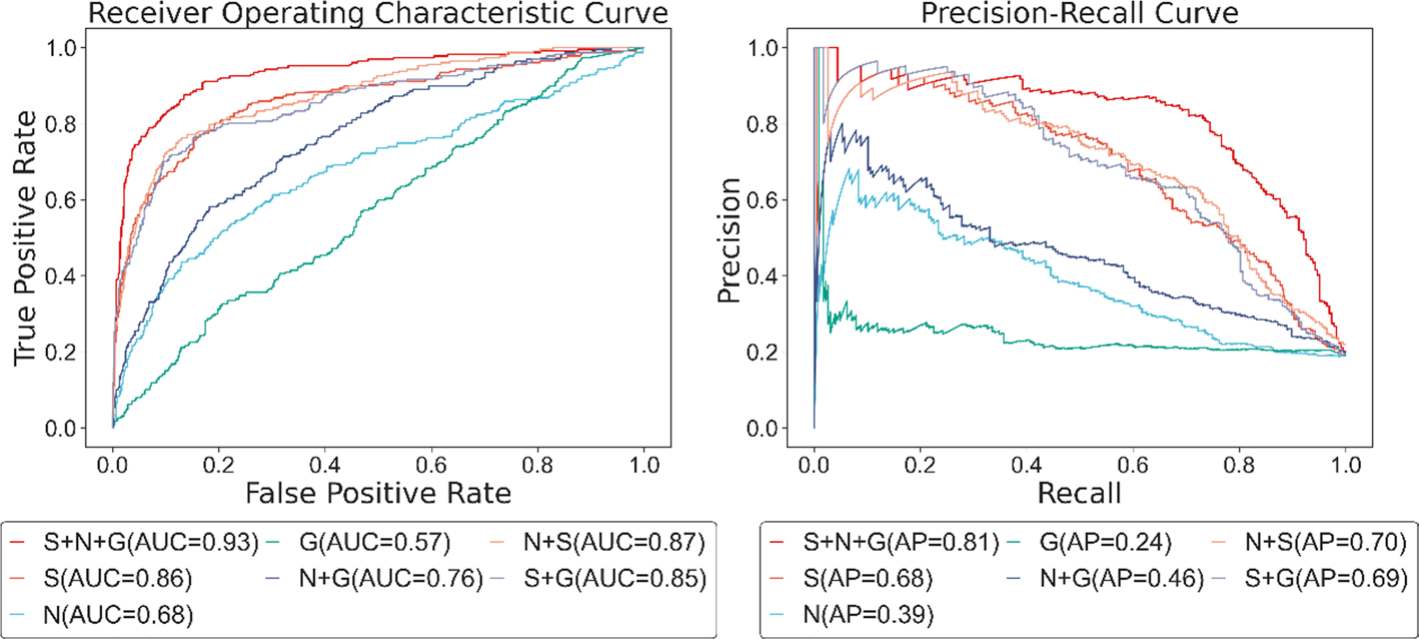
ROC and PR curve of different feature combinations. *S* subcellular localization features, *N* network embedding features, *G* gene expression profile features, *N* + *G* network embedding features plus gene expression profile features, *N* + *S* network embedding features plus subcellular localization features, *S* + *G* subcellular localization features plus gene expression profile features, *S* + *N* + *G* subcellular localization features plus network embedding features and gene expression profile features

Figure 8 plots the ROC and PR curves of the different components. In terms of the performance of the single feature, the subcellular localization with an AUC score of 0.86 and AP score of 0.68 is significantly higher than the network embedding feature and the gene expression features with AUC scores of 0.68, 0.57, and AP scores of 0.39, 0.24, which indicates that subcellular localization feature contributes the most in the prediction task. From the perspective of the combination among features, the addition of subcellular localization features on network embedding features and gene expression features distinctly improves the capability with an AUC score of 0.19, 0.28, and an AP score of 0.41, 0.45 higher than the original ones. Albeit the network embedding features helps little on subcellular localization features, when incorporated with gene expression profiles feature, it raises the AUC score and AP score by 0.21 and 0.22 from the previous. Nonetheless, the participation of gene expression features hardly promotes the performance of other features. Not to mention that the AUC score of subcellular localization features decrease by 0.01 when combined with it. Yet, based on network embedding features and subcellular localization features, the engagement of gene expression profiles enhances the AUC score from 0.87 to 0.93 and AP scores from 0.70 to 0.81, which proves its function to a certain extent.

Table 4 tells the results of these combinations on other metrics. Remarkably, the recall of gene expression features is 0.0396 and 0.4449 higher than the subcellular localization feature and network embedding feature. Furthermore, the addition of gene expression data on the network embedding feature reduces the accuracy and precision by 0.0234 and 0.061, while recall increases by 0.2599, which might be an embodiment of the trade-off between the metrics and the features. Thus, we demand to improve other indicators while maintaining the fine result of existing metrics. Ultimately, adding the gene expression profiles on the combination of network embedding features and subcellular localization features boosts the accuracy, precision, recall, f1 score, sensitivity, and NPV by 0.0325, 0.0593, 0.1497, 0.1039, 0.0051, and 0.0331 respectively, which manifests that our method (N + S + G) realized an excellent balance on the three biological features.

**Table 4:** The performance of different feature combinations

Method	Accuracy	Precision	Recall	F1 score	Sensitivity	NPV
S + N + G	0.9048	0.7306	0.7885	0.7585	0.9320	0.9496
N	0.8122	0.5094	0.2379	0.3243	0.9464	0.8416
S	0.8639	0.6404	0.6432	0.6418	0.9156	0.9165
G	0.4566	0.2112	0.6828	0.3226	0.4037	0.8448
N + G	0.7888	0.4484	0.4978	0.4718	0.8568	0.8795
N + S	0.8723	0.6713	0.6388	0.6546	0.9269	0.9165
S + G	0.8673	0.6560	0.6300	0.6427	0.9228	0.9143

*N* network embedding features, *G* gene expression profile features, *N* + *G* network embedding features plus gene expression profile features, *N* + *S* network embedding features plus subcellular localization features, *S* + *G* subcellular localization features plus gene expression profile features, *S* + *N* + *G* subcellular localization features plus network embedding features and gene expression profile features

The poor performance of gene expression profiles as a single component comes as no surprise due to the utilization of gene expression profiles being mostly implemented on only one gene series matrix. GSE7645, employed in our model, stores the microarray data of the temporal response of the yeast to oxidative stress, in which every protein corresponds to 48 expression data. Thus, it is unfair to count on a single gene expression matrix from one specific experiment to comprehensively measure every node in a network. Comparatively speaking, PPI network and subcellular localization data are collected and integrated from multiple databases by researchers, which conclude various experimental results, numerous pieces of literature, and extensive annotation information. The quality, quantity, and dimensionality of two biological information cannot be compared with simple series data from one experiment, and it is natural for them to perform better. However, in general, the inclusion of gene expression data improved the performance of the model in all aspects.

### Comparison of disposals on the unbalanced dataset

As previously mentioned, essential proteins merely take up a minor proportion in all samples. Taking yeast data used in our experiments as an example, the ratio of essential proteins is 18.90%. Therefore, the unbalanced dataset is also a problem to be considered. Referring to the means in DeepEP [25], we applied a sampling method in our training. Supposing the numbers of essential proteins and non-essential proteins are E and NE, in every iteration, we randomly select E non-essential proteins and combine them with all essential proteins to form a training set.

15 $$p1 = \left( {1 - E/NE} \right)^{n}$$

To alleviate the problem that some non-essential proteins may not be capable of participating in training, we iterate the process 20 times and randomly pick negative samples at every turn. The p1 (15), which indicates the probability of one non-essential protein not being chosen in the training process with the sampling method applied is approximately 0.00496, where n represents the number of epochs.

16 $$p2 = \left( {1 - 2 \cdot E/\left( {E + NE} \right)} \right)^{n}$$

Next step, we use the raw dataset for comparative study. To minimize the differences in other configurations, we arbitrarily sample 2 × E data as a train set to keep the same size as the balanced dataset. When repeating 20 times, the probability, p2 (16), of one protein not being selected is nearly 7.5e−05. Many studies have also applied class weight or sample weight to deal with the unbalanced learning problem. Similarly, we also conducted experiments to compare class weight and sampling methods. Set the class weight of essential protein, which is the minority class in this experiment, to 1–10, while keeping the class weight of nonessential protein at 1, and then observe the impact of class weight on performance.

**Fig. 9:**
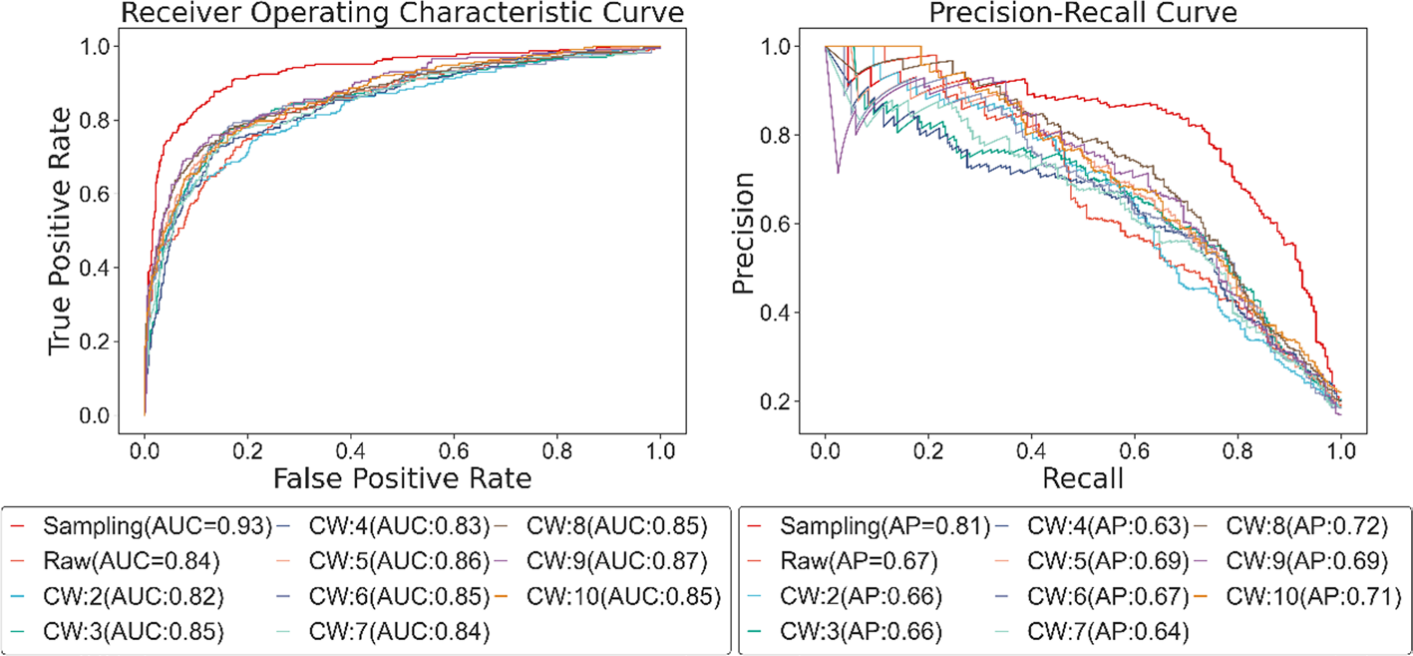
ROC and PR curve of different approaches to the unbalanced dataset. *CW* class weight

As displayed in Fig. 9, the AUC score and AP score of the raw dataset are 0.13 and 0.20 lower than the result of the balanced dataset, which employs the sampling method. Although the performance results vary for different class weights, they all show some improvement over the original dataset. In the process of class weight parameter adjustment, the model obtains the largest AUC score of 0.87 when the class weight is 8 and the largest AP score of 0.72 when the class weight is 9. The worst performance is obtained when the class weight is 4, corresponding to AUC and AP values of 0.83 and 0.63 respectively, which is unexpected. Since the ratio of essential proteins to non-essential proteins in the original dataset is approximately 1:4, a balanced state should be reached when the class weight was 4.

We find no clear pattern in the AUC or AP scores for different class weights, with models performing relatively well when the class weight is 5, 8, 9, and 10, and relatively poorly when the class weight is 2, 3, 4, and 7, so we cannot simply relate the value of class weight to the capability of the model. Furthermore, the difference between these models is not significant, which suggests two things: firstly, class weight, as a means of adjusting for unbalanced datasets, improves model performance to some extent but is not stable and we cannot directly obtain the best class weight value. Further, the model shows robustness to class weight parameter adjustment.

As exhibited in Table 5, the raw dataset, with accuracy, precision, recall, f1 score, specificity, and NPV 6.42%, 14.77%, 24.43%, 19.56%, 2.25%, and 5.40% lower than the balanced dataset, strongly proves the necessity of applying balanced dataset. Furthermore, we find that with the increase of class weight, the recall value of the model shows an upward trend, while the precision value gradually decreases, and the values of accuracy and F1 score are unstable. Class weight is a way to weight the loss function in training. In our test, more and more essential proteins and non-essential proteins are judged as essential proteins. In this case, we can discover more essential proteins, but in the meantime, we also misjudge a large number of non-essential proteins as positive samples, which is the reason for the increase in recall value and the decrease in precision value. Finally, we draw two conclusions. First, the problem of the unbalanced dataset is one of the concerns that need to be taken into account when identifying essential proteins. Second, the sampling method is more effective and stable than the class weight when solving unbalanced datasets.

**Table 5:** The performance of different approaches to the unbalanced dataset

Method	Accuracy	Precision	Recall	F1 score	Specificity	NPV
Sampling	0.9048	0.7306	0.7885	0.7585	0.9320	0.9496
Raw	0.8406	0.5829	0.5442	0.5629	0.9095	0.8956
CW = 2	0.8689	0.7379	0.4735	0.5768	0.9609	0.8870
CW = 3	0.8431	0.5870	0.6864	0.6328	0.8815	0.9197
CW = 4	0.8489	0.6266	0.6083	0.6173	0.9092	0.9026
CW = 5	0.8523	0.5887	0.6964	0.6380	0.8881	0.9271
CW = 6	0.8589	0.6244	0.5991	0.6115	0.9180	0.9096
CW = 7	0.8406	0.5596	0.6920	0.6188	0.8747	0.9251
CW = 8	0.8648	0.6522	0.6904	0.6707	0.9082	0.9217
CW = 9	0.8673	0.5902	0.7094	0.6443	0.8995	0.9382
CW = 10	0.8489	0.6069	0.6709	0.6373	0.8928	0.9167

### Comparison with traditional centrality methods

To compare the performance with other methods, we conduct ten centrality methods on the same PPI network. DC, BC, EC, CC, and SC, are realized based on the package NetworkX, while SoECC, MNC, ClusterC, LAC, and LID are implemented on the theory proposed by researchers. Same as common experimental procedures to measure the performance in centrality methods, first, we calculate the centrality values of every protein in the PPI network. Secondly, the proteins are ranked in descending order based on the values. Then, the top-ranked 19% of proteins are selected as candidate essential proteins. Last, based on this partition, we calculate evaluation metrics according to the true labels of proteins.

**Fig. 10:**
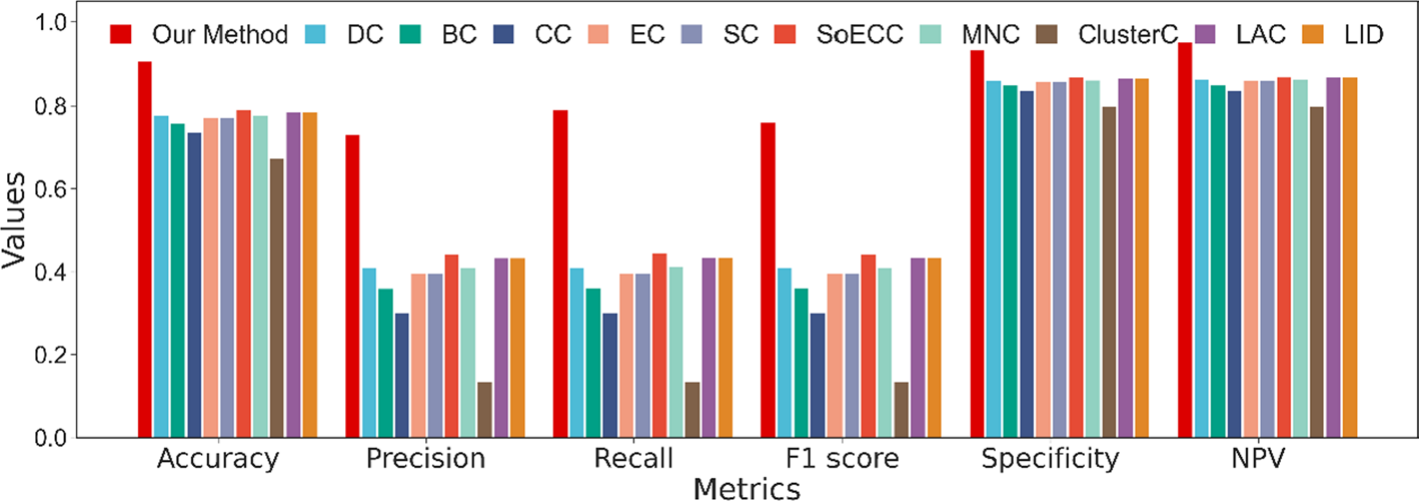
Performance of our method and centrality methods

From Fig. 10, our method significantly outperforms the centrality methods with accuracy, precision, recall, and f1 score. The results of the comparison are as we expected. Our model takes three inputs and uses different treatments to extract patterns of features, whereas traditional centrality methods predict essential proteins based on only one of these inputs, the PPI network. With the increase in network scale and information noise, it is inadequate to capture the topological properties with one single scalar. However, it is unnecessary to completely deny the centrality method for every proposed theory is based on rigorous theoretical derivation and real-world data testing. Thus, if we abandon the primitive measurement procedures, conversely regarding the centrality index as a characteristic of a protein, and put it into deep learning, that would take a specific effect.

### Comparison with machine learning methods

Machine learning methods have been widely applied to identify essential proteins. To demonstrate the superiority of our proposed method, we conduct several comparison experiments with six traditional machine learning methods and one deep learning method, DeepEP. Firstly, Support Vector Machine (SVM), AdaBoost, Logistic Regression (LR), Naive Bayes (NB), Random Forest (RF), and Decision Tree (DT), implemented by the scikit-learn package with default parameters, are tested on the same inputs as our model. For shallow machine learning algorithms, the training and testing datasets are divided in a ratio of 8:2. We also use the sampling method to provide a balanced dataset during the training. Furthermore, DeepEP, a deep learning model to identify essential proteins, which employs node2vec to learn network representations and multi-scale CNN to extract features from gene expression profiles, is tested on our dataset. With the other parameters remaining the same, we modified the values of the two parameters, time step and channel, in DeepEP to fit the shape of our features, as different gene series matrices were used in the two studies. To ensure the fairness of comparison, we use the same data as our model. For the compared machine learning algorithm, we use the same PPI network, gene expression profile, and subcellular localization. For the compared deep learning methods, we use the same PPI network and gene expression profile due to the different construction of the models.

**Fig. 11:**
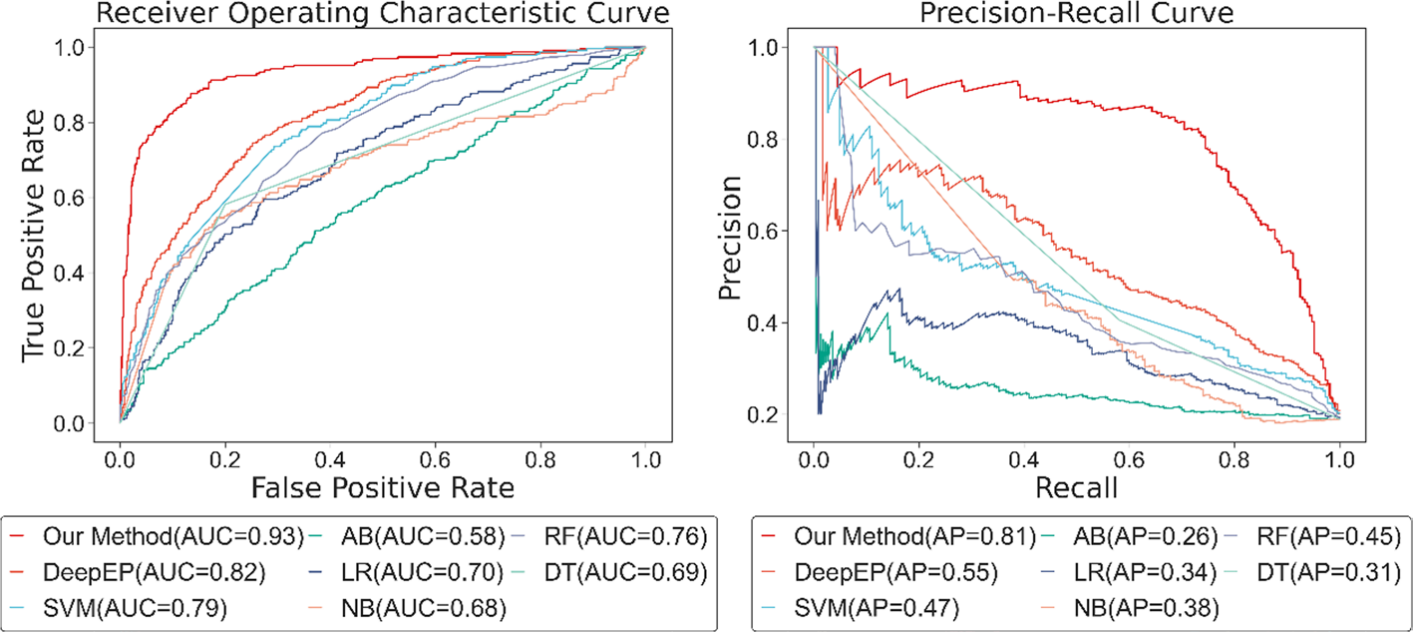
ROC and PR curve of our method and other machine learning methods. *SVM* support vector machine, *LR* logistic regression, *NB* naive bayes, *RF* random forest, *DT* decision tree

Figure 11 plots the ROC and PR curves of our method and other machine learning methods. The AUC score of our method is 0.11, 0.14, 0.35, 0.23, 0.35, 0.17, and 0.24 higher than that of DeepEP, SVM, AdaBoost, Logistic Regression, Naive Bayes, Random Forest, and Decision Tree. Correspondingly, the AP score of our method is 0.26, 0.34, 0.55, 0.47, 0.43, 0.36, and 0.50 exceeds that of DeepEP, SVM, AdaBoost, and Logistic Regression, Naive Bayes, Random Forest, and Decision Tree. From the trend of the curves, we can see that deep learning algorithms generally outperform shallow machine learning algorithms, and relatively speaking, among the shallow machine learning methods SVM has the best adaptability for this classification problem.

Table 6 displays other metrics on these methods. Compared with DeepEP, our method is 14.19%, 28.56%, 10.54%, 21.95%, 14.88%, and 4.29% higher than DeepEP in accuracy, precision, recall, f1 score, specificity, and NPV, which confirms the assistance of subcellular localization data on classification tasks. The superior capability of two deep learning methods to other machine learning on most metrics also demonstrates that deep learning recognizes more patterns than machine learning. Adaboost and Random Forest, with low precision of 0.2597, 0.2051, and low recall of 0.2643, 0.2819, reveal a poor performance on such task. Although Naive Bayes and Logistic Regression show a high recall of 0.8150, and 0.8767, their accuracy, precision, and f1 score are inferior, which indicates the two methods classify many non-essential proteins as essential proteins, which is undesired in our experiments. While Decision Tree and SVM present a relatively better performance among other machine learning methods, they are unable to compete with deep learning methods.

**Table 6:** The performance of our method and other machine learning method

Method	Accuracy	Precision	Recall	F1 score	Specificity	NPV
Our method	0.9048	0.7306	0.7885	0.7585	0.9320	0.9496
DeepEP	0.7629	0.4450	0.6831	0.5390	0.7832	0.9067
AdaBoost	0.7179	0.2597	0.2643	0.2620	0.8239	0.8273
Decision Tree	0.7588	0.4049	0.5815	0.4774	0.8002	0.8911
Naive Bayes	0.4766	0.2403	0.8150	0.3711	0.3975	0.9019
Logistic Regression	0.4332	0.2341	0.8767	0.3695	0.3296	0.9195
Random Forest	0.6569	0.2051	0.2819	0.2375	0.7446	0.8160
SVM	0.7905	0.4545	0.5286	0.4888	0.8517	0.8854

### Test on Homo sapiens data

Since essential proteins are crucial for the understanding of the minimal requirements for cellular life, which is important for the discovery of human disease genes and defending against human pathogens, it is more meaningful to utilize the human protein interactome to find the essential protein candidates. Therefore, we further conduct experiments on the data of Homo sapiens.

PPI network (BioGRID, Release 4.4.200, July 2021) and subcellular localization data (COMPARTMENTS, October 2021) of Homo sapiens are downloaded from the same version as Saccharomyces cerevisiae. The human PPI network contains 537,790 edges and 19,093 nodes after removing repetitive edges and self-loops, with an average degree of 28.17. As for subcellular localization data, we apply the same disposal as yeast data, rank the subcellular localization according to the number of proteins they correspond to, and select the top 1024 ones, covering 93.21% of proteins in the PPI network. For the extraction of essential proteins, drawing lessons from the approach in DeepHE [22], we download a total of 20 subsets of all human essential proteins in the DEG database and consider a protein as vital if it appears in more than 5 subsets. There are 2161 essential proteins after processed, taking up 11.32% of proteins in the PPI network.

Gene expression data, GSE41828 [43], is retrieved from the GEO database as well. GSE41828 contains the expression data of TWEAK-treated time course in U2OS cells, which aims to explore the role of TWEAK in tumor growth and antitumor immune response and the activity and mechanism of RG7212, an antagonistic anti-TWEAK antibody, in tumor models. The experiment set five timesteps, five replicate samples, and two experimental conditions, TWEAK treated and Untreated. The raw series matrix file held 54,675 probe records, leaving 43,138 entries after matching probe ids from the annotation package. After removing duplicate entries, the matrix owns 20,857 records, in which 16,926 proteins exist in the PPI network, covering 88.65% of the total sample.

**Fig. 12:**
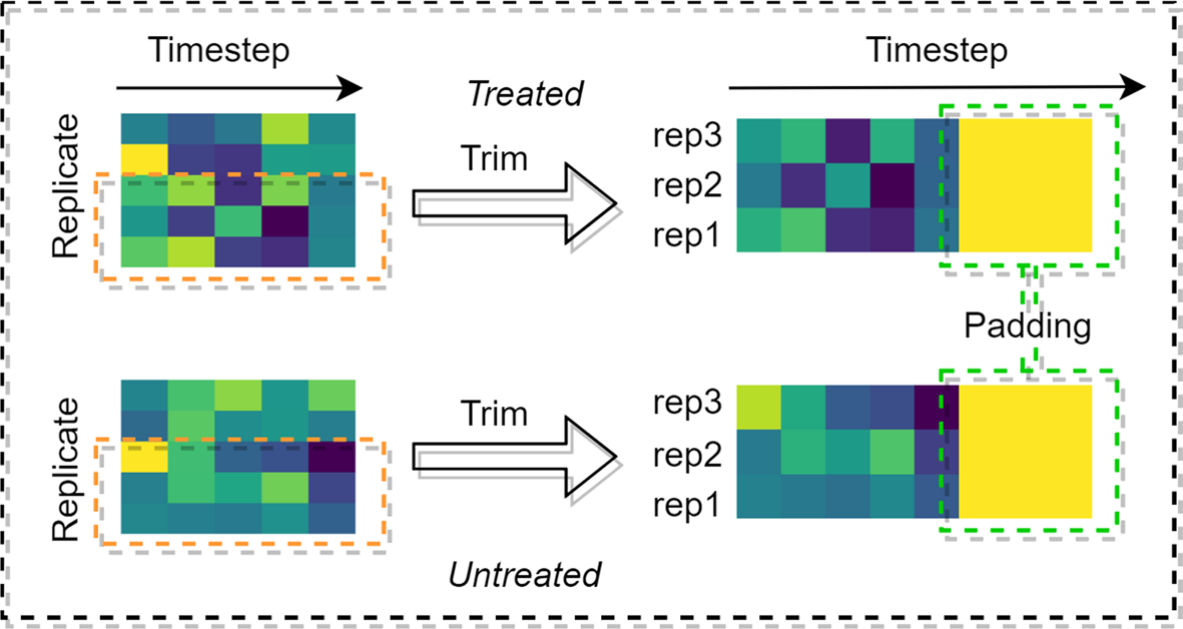
The process of GSE41828

Figure 12 displays the process of gene expression data of Homo sapiens. To fit the input shape of the gene expression module in our model, we trim and pad every data in GSE41828, selecting rep1, rep2, and rep3 as replicate samples. Considering the original data only owns five timesteps, we pad the timestep with zeros. To ensure the fairness of the experiment, we use the same PPI network, subcellular localization, and gene expression data as the input in the comparison among our proposed model, DeepEP, and shallow machine learning methods. For the supervised learning methods, we split the original dataset, of which 80% is used as the training set and 20% as the test set, which is not involved in any training process. The hyperparameters, input shape, and other settings of the model remain the same as those of the yeast experiment. For unsupervised learning methods such as the centrality method, we use the same method as for the yeast data, first calculating the centrality value for each protein, then sorting them in descending order according to the value, and selecting the top 11% of proteins as candidate essential proteins.

**Fig. 13:**
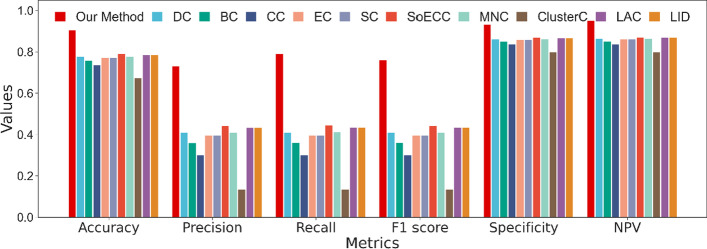
Compare with centrality methods on Homo sapiens data

Figure 13 displays the results of the comparison with traditional centrality methods. Our method outperforms centrality methods with all metrics higher, especially the value of precision, recall, and f1 score. Among traditional centrality methods, LAC and LID perform the best, while EC, SC, and SoECC perform a little worse, but the overall difference is not significant. DC, CC, and BC are mediocre, while ClusterC has the worst results.

Combining the result of Fig. 10, the comparison with centrality methods on yeast PPI network, we find the value of precision, recall, and f1 score of our model have decreased, while the value of accuracy, specificity, and NPV slightly increase. Due to the identification of essential proteins is an unbalanced learning problem and the ratio of essential proteins in the human PPI network is only 11.32%, thus, the metrics as precision, recall, and f1 score are more significant. From an overall perspective, the performance of the model on the human dataset is reduced. Notably, compared to the yeast network, the metrics of centrality methods have increased in all aspects of the human network, mainly accuracy, specificity, and NPV. We believe it is the rise in the number of negative samples in the dataset that has enhanced the capability of negative sample identification, but the main intention of our study is to identify the positive samples. Another noteworthy point is in the yeast network, SoECC presents the best result, while in the human network, LAC and LID outperform other methods. Also, CC gives a poor result in the yeast PPI network, while it surpasses the performance of DC and BC in the network of H. sapiens, which reveals the instability of centrality approaches. The size of the PPI network varies greatly between H. sapiens and S. cerevisiae. The human network has 4.12 and 3.19 times the number of edges and nodes as the yeast network respectively, which makes it denser. It may cause a difference in centrality performance. We also trimmed and padded the gene expression data, which resulted in a loss of data and thus impact the performance of the model.

**Fig. 14:**
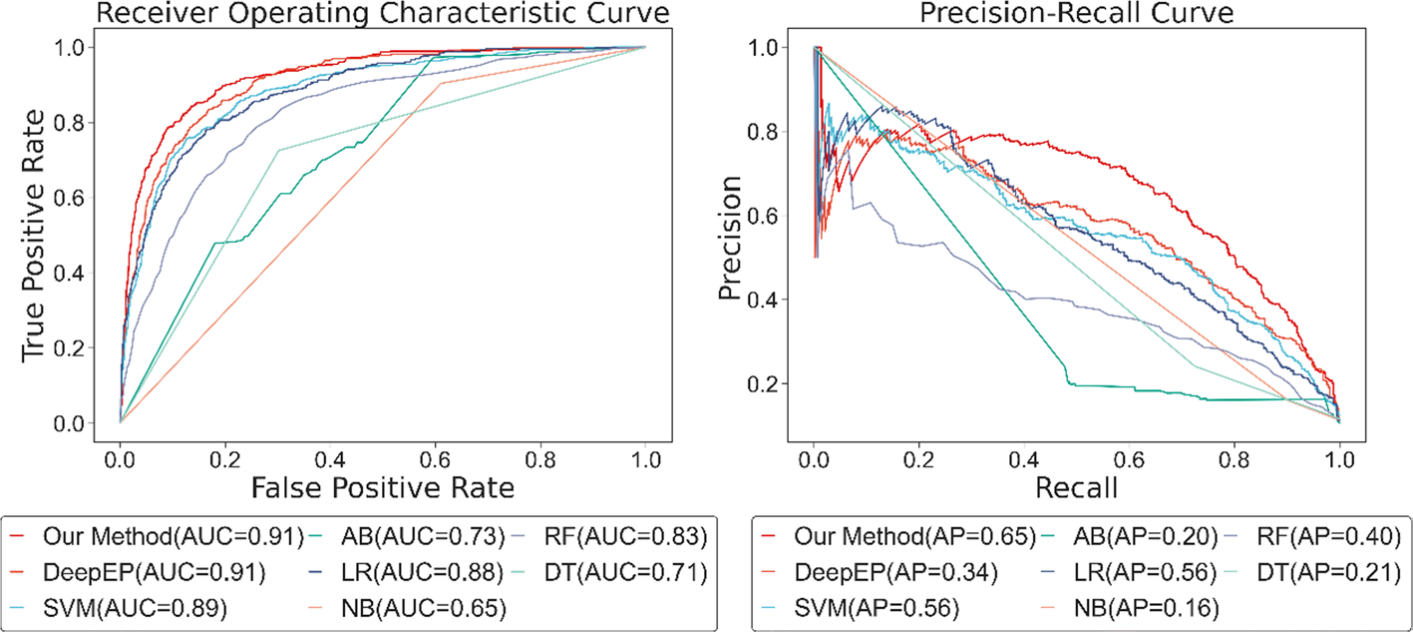
ROC and PR curve of our method and other machine learning methods. *SVM* Support Vector Machine, *AB* AdaBoost, *LR* Logistic Regression, *NB* Naive Bayes, *RF* Random Forest, *DT* Decision Tree

The result of machine learning, DeepEP, and our method is shown in Fig. 14. The AUC score of our model and DeepEP is higher than shallow machine learning methods, and the AP score of our method is higher than other machine learning methods, proving that in the H. sapiens PPI network, our model still owns the best performance.

Nonetheless, combining the results of yeast data in Fig. 11, the AP score and AUC score of our model in human data, which are vital measurements in unbalancing learning, have reduced. The AUC score of DeepEP enhances by 0.09, while its AP score reduces by 0.21. Among shallow machine learning methods, SVM performs best, which is the same as the result of yeast data, and its AUC score and AP score increase by 0.10 and 0.09 separately, proving that SVM suits the task of essential proteins identification best. Logistic Regression shows normal ability in the yeast data, but its capability improves greatly in human data, with AP score and AUC score raise 0.18 and 0.22. The overall performance of the Decision Tree on yeast data is not poor, but it presents undesirable results on the human data. Naive Bayes and AdaBoost still reveal unacceptable outcomes on the data of human, which is identical to the conclusion of yeast data, demonstrating that these two approaches are not applicable for such a problem.

It is worth mentioning that, although the AUC score of DeepEP is high, its AP score is lower than SVM, logistic regression, and random forest. We infer that there may be some main reasons for this result as follows. First, DeepEP is designed to receive only two biological information as inputs, gene expression profiles and PPI network, lacking inputs for subcellular localization compared to shallow machine learning methods, which proves that subcellular localization may be more important in the identification of key human proteins. In addition, we have trimmed the human gene expression profile data, resulting in some missing information, and using the expression data corresponding to the experimental results to explain the proteins of the entire PPI network may not be comprehensive.

We also tried to use the trainable parameters obtained from S. cerevisiae to predict the essential proteins of humans, but the result is not satisfactory. Because genomic differences between species are significant, also, the two gene expression data correspond to completely different experiments, and the patterns they learn are certainly different. We have to trim and pad the gene expression chosen for humans to fit the shape of the input, which causes the loss of some information. All these problems led to bad results. We admit it is the limitation of our model and hope that future work will have a solution to this problem.

## Conclusion

We proposed a deep learning framework for identifying essential proteins. There are three main purposes of our study: First, we want to explore whether the treatment we perform on each element is preferable and the contribution of each element to the model prediction. Secondly, we want to find a better way to address unbalanced datasets. Ultimately, we want to know if the proposed model performs better than other methods in predicting essential proteins.

For gene expression profiles, we demonstrate that separating the experiment environments as different channels and extracting their features along the time step is better than using ordinary convolution. Concerning subcellular localization, we indicate that the result of applying the long vector from the integrated channel as the feature space is significantly excellent compared to the frequently-used 11-dimensional vector obtained from the knowledge channel. When handling the PPI network, the embedding features generated by the node2vec technique are preferable to centrality indexes. Subcellular localization information plays the most vital role in the classification task, followed by the network embedding features, and gene expression profiles take a comparatively less effect. In addition, we examined the impact of unbalanced datasets and confirmed that the sampling method is an effective way to address this type of problem. To prove the capability of our method, we compare our model with ten centrality methods, six machine learning algorithms, and one deep learning model. Results show that our proposed approach is better than the existing methods. We further test our model on the data of Homo sapiens. Although our model still outperforms other methods, the metrics are not as desirable as the yeast data. Results of directly applying training parameters of S. cerevisiae to predict the essential proteins of H. sapiens are also unacceptable. The difference between the genomes of the two species is considered to be the main obstacle. Addressing the limitations of deep learning on this problem is also one of the concerns in the future.

In future work, we will integrate more gene expression data to explore multi-aspect patterns. Since the gap between the performance of the node2vec embedding features and the traditional centrality feature is trivial, we are interested in a more applicable technique to extract the topological properties of the PPI network. Besides, our experiment uses quite a long vector to represent the subcellular localization, which consumes computational costs, so a more sophisticated treatment for subcellular localization information is required.

## Data Availability

The source code and processed dataset of our model are available at (https://github.com/LionKingAHAU/MBIEP). PPI network is downloaded from BioGRID (https://downloads.thebiogrid.org/BioGRID). Gene expression profile is retrieved from GEO (accession number: GSE7645). Subcellular localization data is obtained from the "all channel integrated" column of yeast from COMPARTMENTS (https://compartments.jensenlab.org/Search). The essential proteins are downloaded from OGEE (https://v3.ogee.info/#/home). Notably, when entering the page, it may indicate that the site's security certificate is invalid and that you need to turn off the security warning and use an unsecured connection. We also download essential proteins from DEG (http://origin.tubic.org/deg/public/index.php).

## Abbreviations

CNN: Convolutional neural network
RNN: Recurrent neural network
SVM-RFE: Support vector machine-recursive feature elimination
CHP: Cumene hydroperoxide
AUC: Area under the curve
AP: Average precision
PR: Precision-recall
ROC: Receiver operating characteristic
